# A homogeneous dopamine–silver nanocomposite coating: striking a balance between the antibacterial ability and cytocompatibility of dental implants

**DOI:** 10.1093/rb/rbac082

**Published:** 2022-10-20

**Authors:** Shuang Wang, Zichen Wu, Yankai Wang, Huilei Hong, Lijie Zhang, Zhaoyang Chen, Pengkang Zhang, Zirui Chen, Weibo Zhang, Shunli Zheng, Quanli Li, Wei Li, Xiangyang Li, Hua Qiu, Jialong Chen

**Affiliations:** Key Laboratory of Oral Diseases Research of Anhui Province, Stomatologic Hospital and College, Anhui Medical University, Hefei, Anhui 230032, China; Key Laboratory of Oral Diseases Research of Anhui Province, Stomatologic Hospital and College, Anhui Medical University, Hefei, Anhui 230032, China; Key Laboratory of Oral Diseases Research of Anhui Province, Stomatologic Hospital and College, Anhui Medical University, Hefei, Anhui 230032, China; Key Laboratory of Oral Diseases Research of Anhui Province, Stomatologic Hospital and College, Anhui Medical University, Hefei, Anhui 230032, China; Key Laboratory of Oral Diseases Research of Anhui Province, Stomatologic Hospital and College, Anhui Medical University, Hefei, Anhui 230032, China; Key Laboratory of Oral Diseases Research of Anhui Province, Stomatologic Hospital and College, Anhui Medical University, Hefei, Anhui 230032, China; Key Laboratory of Oral Diseases Research of Anhui Province, Stomatologic Hospital and College, Anhui Medical University, Hefei, Anhui 230032, China; Key Laboratory of Oral Diseases Research of Anhui Province, Stomatologic Hospital and College, Anhui Medical University, Hefei, Anhui 230032, China; Key Laboratory of Oral Diseases Research of Anhui Province, Stomatologic Hospital and College, Anhui Medical University, Hefei, Anhui 230032, China; Key Laboratory of Oral Diseases Research of Anhui Province, Stomatologic Hospital and College, Anhui Medical University, Hefei, Anhui 230032, China; Key Laboratory of Oral Diseases Research of Anhui Province, Stomatologic Hospital and College, Anhui Medical University, Hefei, Anhui 230032, China; Key Laboratory of Oral Diseases Research of Anhui Province, Stomatologic Hospital and College, Anhui Medical University, Hefei, Anhui 230032, China; Key Laboratory of Oral Diseases Research of Anhui Province, Stomatologic Hospital and College, Anhui Medical University, Hefei, Anhui 230032, China; Key Laboratory of Oral Diseases Research of Anhui Province, Stomatologic Hospital and College, Anhui Medical University, Hefei, Anhui 230032, China; Key Laboratory of Oral Diseases Research of Anhui Province, Stomatologic Hospital and College, Anhui Medical University, Hefei, Anhui 230032, China

**Keywords:** titanium implants, antibacterial, cytocompatibility, silver, nanocomposites

## Abstract

Silver has been widely used for surface modification to prevent implant-associated infections. However, the inherent cytotoxicity of silver greatly limited the scope of its clinical applications. The construction of surfaces with both good antibacterial properties and favorable cytocompatibility still remains a challenge. In this study, a structurally homogeneous dopamine–silver (DA/Ag) nanocomposite was fabricated on the implant surface to balance the antibacterial activity and cytocompatibility of the implant. The results show that the DA/Ag nanocomposites prepared under the acidic conditions (pH = 4) on the titanium surface are homogeneous with higher Ag^+^ content, while an obvious core (AgNPs)–shell (PDA) structure is formed under neutral (pH = 7) and alkaline conditions (pH = 10), and the subsequent heat treatment enhanced the stability of PDA–AgNPs nanocomposite coatings on porous titanium. The antibacterial test, cytotoxicity test, hypodermic implantation and osteogenesis test revealed that the homogeneous PDA–AgNPs nanocomposite coating achieved the balance between the antibacterial ability and cytocompatibility, and had the best outcomes for soft tissue healing and bone formation around the implants. This study provides a facile strategy for preparing silver-loaded surfaces with both good antibacterial effect and favorable cytocompatibility, which is expected to further improve the therapeutic efficacy of silver composite-coated dental implants.

## Graphical Abstract

**Figure:**
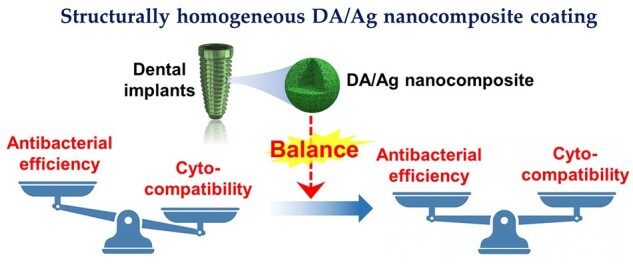

## Introduction

Titanium dental implant has become a routine treatment to patients with dentition defect and dentition loss [1]. However, failures can still happen to implants in spite of their high survival rate and good stability [2]. Both the contamination-related early failure [3] and the peri-implant infections related late failure [4] reveal that microbes are the main issue of implant failure. Dental implants are more predisposed to infection compared to natural teeth on account of their histological and immunological differences [5]. Bacteria could colonize on the implant within 30 min after implant surgery [6] and then give rise to the formation of structural and functionally organized biofilm [5, 7]. Biofilm is the major etiological factor of peri-implant mucositis and peri-implantitis [8].

Surface antibacterial modification is considered to be an effective way to prevent peri-implant infection and has good application prospects [9]. Antibiotics, peptides, metal ions and nanoparticles (NPs) are widely studied and proved to be effective antimicrobial agents [10, 11]. Among them, silver ions (Ag^+^) or silver nanoparticles (AgNPs) have broad-spectrum antibacterial, strong and long-lasting antibacterial activity, especially anti-biofilm properties, and are very promising surface modification additives for implants [12, 13]. The deposition or doping of AgNPs by reducing silver ions on titanium-based substrates based on different reduction systems has been investigated [14–18]. The mussel-inspired dopamine (DA) is a compelling candidate with great potential for building multifunctional platforms due to its chemical diversity [19, 20]. With its redox and metal cation sequestering characteristics, polydopamine (PDA) has the ability to couple with metal ions and reduce them to nano-silver in alkaline solution and finally form the polydopamine–silver nanoparticles (PDA–AgNPs) [21]. Therefore, PDA can play multiple roles as a fixative, reservoir and stabilizer for silver.

Physiological properties of silver-based coatings are closely related to the coating structure. However, the fabrication of structurally diverse PDA–AgNPs coatings still faces challenges. In fact, many researchers have adopted a two-step method to construct PDA–AgNPs coatings, i.e. the PDA coating is first prepared on the substrate, and then the AgNPs are formed on it [17, 22, 23]. This method leaves the silver completely exposed on the outermost surface and cannot avoid direct contact of large amounts of silver with cells or tissues, resulting in severe cytotoxicity. In view of this, some methods to avoid direct exposure of silver are proposed. For example, silver and DA were directly mixed in solution to form a typical core–shell nanostructure, and the DA shell prevented the AgNPs core from being directly exposed to the environment [24–26]. However, applying PDA–AgNPs to device surfaces still faces two major challenges. One is how to make PDA–AgNPs firmly bonded to the surface, which is important to resist frictional shedding during implantation, and the other is how to regulate the nanostructure of PDA–AgNPs to prevent the DA shell from being too thick to prevent the release of silver and thereby sacrificing the antibacterial ability, or too thin to expose the AgNPs and thus exert an obvious cytotoxicity. Therefore, we proposed the *in situ* construction of structurally homogeneous PDA–Ag nanocomposite coating. PDA–AgNPs could be *in situ* formed on the rough porous titanium surface and could effectively bind with the substrate through the catechol–titanium chelating [27]. On the other hand, the homogeneous that could effectively store silver and have an appropriate release rate of silver, through which could minimize the toxicity to cells while being antibacterial, eventually obtain the balance between antibacterial efficiency and cytocompatibility.

Previous studies have demonstrated the reaction pH is one of the crucial factors to tailor the structure of the formed PDA–AgNP [28, 29]. However, most of the studies of fabrication of nano-silver by phenol were carried out under alkaline conditions. Because the alkaline (pH > 7.5) and aerobic condition could accelerate the spontaneous self-polymerization of DA, while the acidic condition did it conversely [30]. The conversion of Ag^+^ into Ag^0^ by the reductive phenol groups of DA also depended on the reaction pH [31]. Besides, the pH would also greatly impact on the properties of AgNPs including the size, shape, aggregation, stability, oxidative dissolution, etc. [32], which could further influence the release behavior and its antibacterial performance and cytotoxicity [33].

In this study, we explored the influence of reaction pH on the structure of PDA–AgNPs nanocomposite, and screen out an optimized condition to obtain the expected structurally homogeneous nanocomposite. In addition, heat treatment was carried out after *in situ* deposition of PDA–AgNPs on porous titanium surfaces to enhance the intra-particle cohesion and inter-particle interactions [34, 35], resulting in more stable nanocomposite coatings. The antibacterial ability and cytocompatibility of the stable PDA–AgNPs coatings was determined through *Staphylococcus aureus* inhibition test and osteoblast-like MC3T3-E1 cells test, respectively. Osteogenesis test on various nanocomposite coatings was further carried out to validate the better outcomes of the homogenous PDA–AgNPs coating. We hope that our strategy could provide a useful reference for the preparation of stable and homogenous silver-loaded nanocomposite coatings, which can effectively prevent peri-implant infections and without sacrificing the osseointegration.

## Materials and methods

### Materials

Commercial pure Ti was purchased from Baoji Non-ferrous Metal Co., Ltd. (Shanxi Province, China). Dopamine hydrochloride (C_8_H_11_NO_2_·HCl), silver nitrate (AgNO3), sodium hydrate (NaOH) and MTT kit were purchased from Sigma-Aldrich (St. Louis, MO). α-Minimum Eagle’s medium (α-MEM), fetal bovine serum and Trypsin–EDTA solution were purchased from Gibco. Ultrapure water from Milli-Q water system was used to prepare the aqueous solutions. The MC3T3-E1 cell line and *S. aureus* ATCC25923 were purchased from the Department of Stomatology affiliated to Shanghai Jiaotong University.

### Preparation of DA/Ag-modified samples

The commercial pure titanium sheets were treated sequentially by mechanical polishing, ultrasonic cleaning and alkali heat treatment as previously reported [18], and the prepared porous titanium sheet was denoted as pTi. AgNO_3_ (0.4 mg/ml) were dissolved in water with a series of pH (pH=4, 7 and 10), respectively. Mixing the AgNO_3 _solution with equal volumes of DA solution (4 mg/ml) at the same pH. Then the pTi were immersed in the mixed solution at 37°C for 24 h to coat the PDA–AgNPs nanocomposite on the surface. Half of the samples were taken out and ultrasonically cleaned with deionized water. According to the pH value of the reaction solution, the obtained samples were denoted as DA/Ag_4_, DA/Ag_7_ and DA/Ag_10_, respectively. The remaining samples in the solution were removed and placed in an oven at 150°C for 2 h, followed by the cleaning with deionized water, and these samples were denoted as DA/Ag_4_-H, DA/Ag_7_-H and DA/Ag_10_-H, respectively.

### Characterization of DA/Ag composite and the coated surface

The surface morphology of the samples was observed by scanning electron microscopy (SEM, Hitachi S-4800). X-ray photoelectron spectroscopy (XPS, Thermo ESCALAB 250) was used to characterize the outermost surface chemical composition. Fourier transform infrared spectroscopy (FTIR, Nicolet ST-IR20SX) was used to characterize the surface functional groups. The water contact angle (WCA, Kruus DSA100) was measured with deionized water at room temperature.

To obtain the total mass of silver on the surface, the sample was placed in 1 ml aqua regia for 5 min by ultrasonic vibration, and then 14 ml deionized water was added. The silver concentration in the solution was detected by Inductively Coupled Plasma Mass Spectrometry (ICP-MS, Thermo X Series 2).

The formation process of the DA–Ag composite in the solution was monitored. Transmission electron microscopy (TEM, Hitachi HT-7700) was used to visualize the morphology of the composite in the solution. The zeta potential and particle size of composite were monitored by the size and zeta potential analyzer (Brookhaven, 90Plus PALS).

### Antibacterial assessment

#### In vitro antibacterial assessment

Briefly, *S. aureus* (ATCC25923) were cultivated on Luria-Bertani (LB) agar plate for 24 h at 37°C, then one colony was picked and add into Brain Heart Infusion broth to culture for 24 h at 37°C. The bacterial suspension was diluted to achieve a final concentration of 10^6^ CFU/ml. To test the stability of antibacterial properties of samples, sterilized samples were immersed in physiological saline (PS) for 7 days. Then, sterile samples with or without PS were placed in 24-well plates, followed by the spreading of 50 μl of bacterial suspension at 10^6^ CFU/ml on each surface and cultured for 4 h at 37°C. Then 2 ml LB broth was added to each well to incubate with the samples at 37°C for the following evaluations: (i) after 24 h incubation, the samples were rinsed with phosphate buffer saline (PBS), and stained with the LIVE/DEAD BacLight Bacterial Viability Kit in a dark room [36], and observed using a fluorescence inverted microscope (Leica, Germany). (ii) After 24 h incubation, the samples were rinsed with PBS and transferred to a centrifuge tube with 2 ml PBS. After ultrasonic and vortex processing for 5 min to strip the bacteria on the sample surface, 10 µl of the PBS in each tube was collected and diluted 10 times, then 50 µl diluent were spread on the agar plate. (iii) After 24 h and 48 h incubation, 150 μl of LB broth was taken out from each well to determine bacterial concentration by recording the optical density at 660 nm (OD 660) using a microplate reader. (iv) Each agar plate was evenly coated with 50 μl bacterial suspension, and the titanium samples were placed face down and closely contacted to agar. After 24 h incubation at 37°C, the samples were photographed with a digital camera and the inhibition zone was recorded.

#### In vivo antibacterial assessment

To evaluate the inhibitory effect of as-prepared surfaces on bacteria in soft and hard tissue around implant site, titanium rods (Ф 1.5 mm × 6 mm) and titanium discs (Ф 8 mm × 1 mm) samples with good antibacterial ability were further were evaluated in male SD rats weighing about 200 g. According to the previous report, 10 μl of *S. aureus* suspension at 10^6^ CFU/ml was slowly injected into the bone marrow cavity of SD rats where sterile titanium rods were then inserted [18]. The wound was sealed with sterile bone wax and stiches. After 1 and 7 days of implantation, a part of the titanium rods were taken out and rolled on the agar plate for 24 h, the rest of the titanium rods were placed in 2 ml PBS, ultrasonicated and vortexed for 5 min, and 50 µl of the solution was drawn and spread on the agar plate at 37°C for 24 h. Besides, 10 μl of *S. aureus* suspension at 10^6^ CFU/ml was slowly injected into the subcutaneous sites of SD rats where sterile titanium discs were then inserted. After 1 and 7 days of implantation, samples were removed and placed in 2 ml PBS for 5 min with ultrasonic and vortex processing, then 50 µl of the solution were transferred to the agar plate to culture at 37°C for 24 h.

### Cytocompatibility assessment

Osteoblastic cell line MC3T3-E1 cells were cultured routinely in α-MEM containing 10% fetal calf serum (FCS) at 37°C for 24 h. When cells had reached ∼80% confluence, these were detached using trypsin to harvest the cells for the following evaluations. Sterile samples were placed in 24-well plates and immersed with 1 ml cell suspension at a density of 1 × 10^5^/ml, the medium was renewed every 2 days. After incubation for 1, 3 and 5 days, cell viability of the samples were evaluated by MTT assay. Meanwhile, the samples were taken out and rinsed with PBS, and immersed with 2.5% glutaraldehyde to fix for 4 h. Then, the samples were rinsed with PBS again and stained with YF555-phalloidin for F-actin and DAPI for nuclei for 15 min before immediate observation under the fluorescence microscope.

### Co-culture of bacteria cells

To study the effect of antibacterial surface on cell adhesion in bacterial environment, co-culture of cells and bacteria was performed. Fifty microliters of *S. aureus* suspension (10^6^ CFU/ml) was dropped and spread on each sterile sample. After incubation for 4 h at 37°C, 1 ml cell suspension at a density of 1 × 10^5^/ml was added to each well. After incubation for 1 and 3 days, the samples were rinsed with PBS followed by staining with LIVE/DEAD BacLight Bacterial Viability Kit and then observed under fluorescent microscope.

### *In vivo* biocompatibility evaluation

#### Subcutaneous implantation experiment

SD rats with a body weight of 180 ± 20 g were anesthetized with sodium pentobarbital (pre-prepared to 30 mg/ml with normal saline, and injected with 1 ml/kg according to the body weight of the rats), and then the back skin is incised symmetrically on the left and right sides, followed by the implantation of DA/Ag-modified (with the coated side facing down) or unmodified titanium discs, and 10 μl of *S. aureus* suspension at 10^6^ CFU/ml was injected into the gap between samples surface and tissue. The skin incision was then sutured and a penicillin solution was dripped on the surface to avoid infection. Rats were sacrificed after 1 month of culture, and the tissue with sample discs was excised. After removing the discs, the surrounding soft tissues were embedded in paraffin, sliced and stained with HE for the evaluation of tissue inflammation.

#### Bone formation assessment

To simulate the invasion of bacteria into the implant site after implant placement, 10 μl of *S. aureus* suspension at 10^6^ CFU/ml was slowly injected into the bone marrow cavity and the subcutaneous sites of SD rats, respectively. Then sterile titanium rods were inserted into the bone marrow cavity and sterile titanium discs were inserted into the subcutaneous sites. After 1 month of implantation, samples along with the surrounding tissues were excised and fixed with 4% paraformaldehyde. Then, micro-computed tomography (micro-CT) (SkyScan 1176, Bruker) was used to analyze the bone formation around the titanium rods.

### Statistics

All experiments were performed at least three independent times. All quantitative data are presented as the means ± standard deviations and were compared with one-way ANOVA tests to evaluate statistical significance using SPSS software. After ANOVA, Tukey’s multiple comparisons test was performed to find significant differences between pairs.

## Results and discussion

### Characterization of different samples

**Figure 1.**
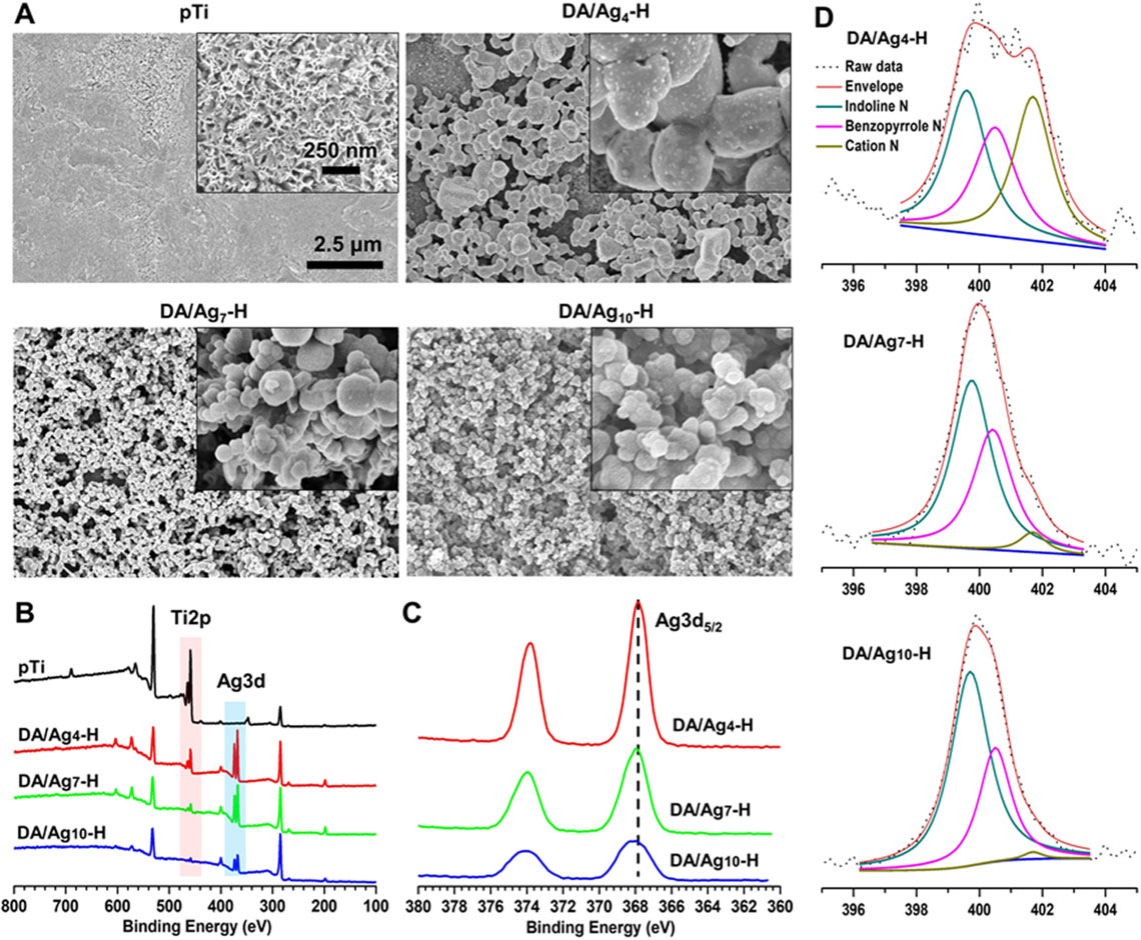
(**A**) Surface morphology of pTi, DA/Ag4-H, DA/Ag7-H and DA/Ag10-H by SEM, and the (**B**) survey XPS spectra of each surface. (**C**) The high-resolution XPS spectra of Ag3d of different samples and (**D**) the fitting peaks of N 1 s on different samples.

The secondary electron image of the surface morphology of different samples is shown in Fig. 1A. Alkaline heat treatment resulted in uniform nanoporous surface topography on pTi to increase specific surface area. After immersion in a DA/Ag solution and then heat treatment, a large number of NPs were coupled with the porous surfaces; in addition, the number of NPs increased and the size of NPs decreased with increasing solution pH from 4 to 10. Surfaces prepared in pH 7 and pH 10 solution were completely coated with NPs. Compared with that of the unheated group (Supplementary Fig. S2A), the number of NPs in the corresponding heated group was more, speculating the reason is that heat treatment could increase the activity of DA to couple with titanium and silver, which makes NPs difficult to peel off the surfaces during ultrasonic cleaning. The fusion degree of NPs in the corresponding heated group was higher than that in the unheated group, which may be triggered by increased self-polymerization of DA at higher temperature [34, 35]. Therefore, heat treatment can make NPs more stable on the porous titanium surfaces.

XPS was used routinely to analyze elemental composition and chemical state within 10 nm of sample coating surfaces [19]. The wide-scan XPS spectrum (Fig. 1B) shows that the Ti 2p and O 1 s peaks are weakened, the C 1 s and N 1 s peaks are increased, and the Ag 3d peak has appeared on the DA/Ag-modified surface compared with the porous titanium surface, indicating that DA and silver successfully deposited on the titanium surface. The atomic concentration of different surfaces was determined by XPS and shown in Table 1. Compared with pTi, the atomic concentration of C, N and Ag increased and that of Ti and O decreased, also indicating the successful preparing of the DA/Ag composite on titanium surfaces. In addition, with the increase of reaction pH, the atomic concentrations of Ti and Ag decreased and that of C and N increased, indicating that the formation of DA/Ag complexes on the surface increased with the increase of reaction pH, and the amount of DA is higher than that of Ag.

**Table 1.** Atomic concentration of different surfaces determined by XPS

Samples	Elements (atom%)				
	C 1s	Ag 3d	N 1s	Ti 2p	O 1s
**pTi**	27.59	0	2.26	20.04	50.11
**DA/Ag_4_-H**	57.97	4.47	4.98	7.22	25.36
**DA/Ag_7_-H**	64.61	3.81	7.04	2.9	21.64
**DA/Ag_10_-H**	69.43	1.82	7.2	1.35	20.2

To analyze the valence state of silver, high-resolution of Ag 3d XPS spectra was performed. As shown in Fig. 1C, the bind energies corresponding to Ag 3d_5/2_ on the DA/Ag_4_-H, DA/Ag_7_-H and DA/Ag_10_-H surfaces were 367.75 eV, 367.83 eV and 367.95 eV, respectively. The higher binding energies of Ag 3d_5/2_ peaks indicated a greater proportion of Ag^0^ existed on the outermost surface, which is consistent with our previous research [17]. This proved that DA had a strong reduction ability in the alkaline environment to convert more Ag^+^ to Ag^0^. In contrast, the acidic reaction condition suppressed such conversion, and resulted in substantial Ag^+^ distribution in DA/Ag_4_-H. We suppose that acidic condition decelerated the oxidation process of DA, decreased the electron transferring to Ag^+^ and finally a mass of monovalence silver ion was immobilized in the DA/Ag_4_-H composite.

To analyze the structure of DA, high-resolution of N 1 s XPS spectra was performed and is shown in Fig. 1D. The products of DA in different stages of oxidation reaction and the chemical state of nitrogen in it is shown in Supplementary Fig. S1. Accordingly, N 1 s spectra can be fitted into three main peaks corresponding to Cation N, Benzopyrrole N and Indoline N with binding energy of 401.6 ev, 400.4 ev and 399.8 ev, respectively. According to the area proportion of fitted peaks in Supplementary Table S1, an increase in the pH value of a solution led to obvious elevation of Indoline N% and reduction of Cation N%, indicating that more DA was deprotonated and then been oxidized to DA-quinone with increasing solution pH, which rearrange to leukodopaminechrome (with Indoline N) through intramolecular cyclization. Subsequent oxidation and rearrangement formed the 5,6-dihydroxyindole (with Benzopyrrole N), and it was further covalently crosslinked to form the final products of polydopamine [19]. However, the acidic environment facilitates the protonation of amine groups on DA, meanwhile suppressing the ionization of phenol groups on DA, thus leading to more Cation N and lower rate of DA polymerization [37]. Besides, as shown in Supplementary Fig. S2B and Supplementary Table S1, Cation N% of the heated group is significantly lower than that of corresponding unheated group. This may be because exogenous oxygen promotes further oxidation of uncycled DA oligomers in high temperature environment, which consumes protons in the coating at the same time [35].

**Figure 2.**
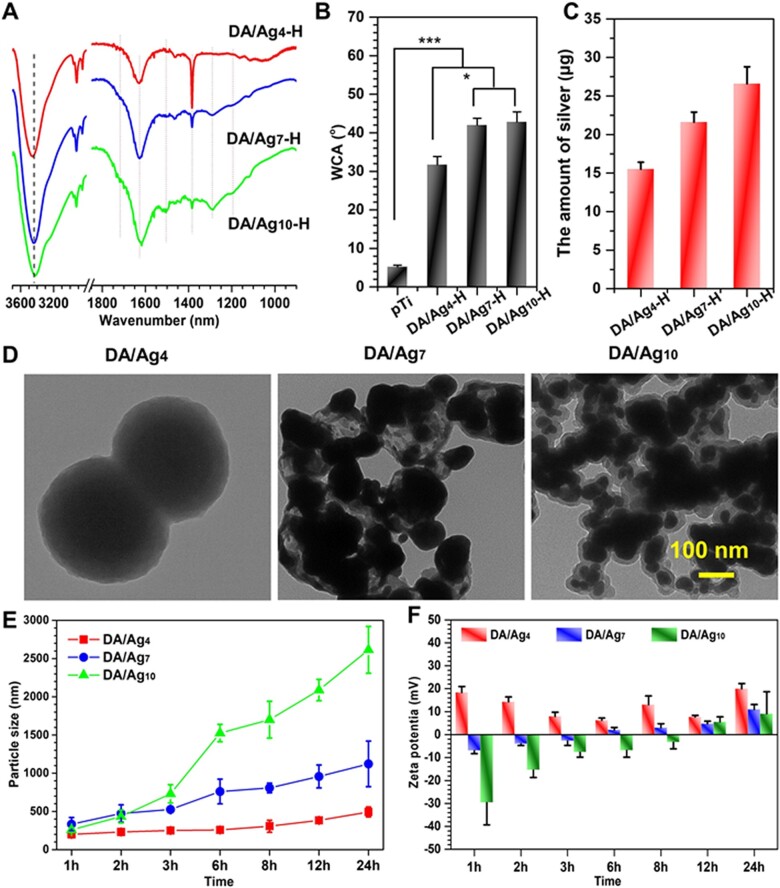
(**A**) The FTIR spectra of DA/Ag4-H, DA/Ag7-H and DA/Ag10-H, (**B**) the WCA of different surfaces and (**C**) the total silver mass of in the coating of different samples. (**D**) The structure of DA/Ag nanocomposites by TEM. (**E**) The particle size evolved over time by DLS. (**F**) ζ-potentia of formed nanoparticles under different pH over time.

The surface components of the DA/Ag-coated samples were scraped off and prepared with KBr pellets, and analyzed its chemical composition by FTIR. As shown in Fig. 2A, the broad absorption band in the 3450–3350 cm^−1^ could be assigned to the stretching of O–H or N–H. This band undergoes a red shift with increasing pH of the solution, speculated that higher pH leading to more quinone components, which could form stronger hydrogen bonding of C = O···H–O or C = O···H–N [38]. The absorbance at 1750 cm^−1^ (C = O stretching vibration) increased with the increasing pH, confirmed that more quinone components were produced at higher pH conditions. DA-quinone, as a precursor molecule for DA polymerization, forms poly(dopamine) through intramolecular cyclization and self-polymerization. The acidic environment results in a low rate of DA polymerization and cyclization, which is consistent with the analysis of the corresponding high-resolution N1s XPS spectra. The characteristic absorption of DA at 1500 cm^−1^ can be attributed to vibration of π-conjugated systems of benzene ring, and the peak intensity dramatically weakened with the decreasing of pH, even disappeared for the DA/Ag_4_-H group, indicating that benzene ring had a strong interference by silver. We speculated that Ag^+^ concentration is higher under acidic pH, because the conversion of Ag^+^ into Ag^0^ was impeded at acid pH as aforementioned. The high concentration of Ag^+^ bonded to the benzene ring on polydopamine, formed of a σ composite (Ag^+^-π interactions), which led to charge transfer from HOMO of phenol into the empty 5 s orbital of Ag^+^ [39] and eventually affect the resonant peak intensity at 1500 cm^−1^. The characteristic peaks of nitrate ions (1380 cm^−1^) [40] were observed in each coating, but it gradually weakened with increasing pH conditions, and was especially strong in DA/Ag_4_-H. It was speculated that a large number of amino groups on DA was protonated to form –${\text{NH}}_{3}^{+}$ under acidic conditions (the Ag^+^-π may also contributed the positively charged site) and then adsorbed negatively charged species of ${\text{NO}}_{3}^{–}$ through electrostatic attraction. In contrary, higher pH conditions resulted less positive charge to attract nitrate, resulting in a significant decrease in the nitrate ion peaks of the DA/Ag_7_-H and DA/Ag_10_-H. The broad absorption band at 1200 ± 100 cm^−1^ mainly corresponds to the C–O and C–N stretching vibrations. Ag^+^, as a Lewis acid, has affinity with oxygen and nitrogen elements, especially has a strong interaction with amino groups. The Ag^+^, which is widely distributed in the coating under acidic conditions, significantly affected the stretching vibrations of C–O and C–N, resulting in weakened stretching vibration of C–N and C–O in DA/Ag_4_-H. While under neutral and alkaline conditions, most of Ag^+^ was converted into AgNPs, so the influence of Ag^+^ on C–O and C–N was greatly reduced, thus the absorption band of 1200 ± 100 cm^−1^ in DA/Ag_4_-H and DA/Ag_7_-H was still observable, especially in DA/Ag_10_-H.

Surface hydrophilicity could affect biological functions such as protein adsorption and bacterial/cell adhesion. Here, a WCA measurement was used to study the surface hydrophilicity. As shown in Fig. 2B, the surface of alkali-heat treatment was super-hydrophilic; however, the surface hydrophilicity reduced after DA/Ag modification. The WCA values were 5.2 **±** 0.4° for pTi, 31.7 **±** 2.2° for DA/Ag_4_-H, 42.0 **±** 1.8° for DA/Ag_7_-H and 42.8 **±** 2.6° for DA/Ag_10_-H. The results showed that there was no significant difference of hydrophilicity between the DA/Ag_7_-H and DA/Ag_10_-H, but the hydrophilicity of both surfaces was significantly lower than that of the DA/Ag_4_-H. This may be related to the lower degree of oxidation of DA, the protonation of amino groups and the richness of Ag^+^ in DA/Ag_4_-H, which favors the interaction of water molecules with these components, making the surface of DA/Ag_4_-H more hydrophilic. Our previous works demonstrated that the WCA of DA (without silver)-coated porous titanium surface was ∼50° [41], which is the closer to the WCA values of DA/Ag_7_-H and DA/Ag_10_-H, indicated more DA components were exposed on DA/Ag_7_-H and DA/Ag_10_-H when compared to the DA/Ag_4_-H.

ICP-MS is a highly sensitive metal ion detection method. Compared with the limited detection depth of XPS (typically < 10 nm for organic coatings), ICP-MS could determine the total amount of silver on DA/Ag-modified samples by quantifying metal ions in the coating pickling solution. As shown in Fig. 2C, the total amounts of silver were 15.5 μg for DA/Ag_4_-H, 21.6 μg for DA/Ag_7_-H and 26.6 μg for DA/Ag_10_-H, indicating that the silver content on the samples increased significantly with the increase of pH, which is opposite to the trend of the atomic percentage of silver in the XPS results. The inconsistent results of ICP-MS and XPS further indicated that silver was coated with more DA with increasing pH, which was consistent with previous speculation based on WCA.

To study the stability of the NPs, the DA/Ag-modified samples were immersed in PS for 7 days, and the surface morphology was observed. As shown in Supplementary Fig. S2C, the size of NPs in DA/Ag_4_-H-PS decreased and the exposed substrate area increases, indicating that NPs degrade gradually in solution. The number of small size of particles on the surface of DA/Ag_7_-H-PS increased slightly, but NPs were still completely covered the surface of pTi. DA/Ag_10_-H surface has no obvious change before and after immersion. These results indicated that the stability of the NPs ranked in ascending order were as follows: DA/Ag_10_-H> DA/Ag_7_-H > DA/Ag_4_-H. Immersion test showed that most of the nanocomposite particles prepared under acidic condition became smaller, indicating that their degradation was the fastest, followed by neutral and alkaline.

Because DA/Ag composite adhered to the sample’ surfaces as NPs, TEM was used to investigate the inner structure of DA/Ag NPs prepared in different pH solutions by mass-thickness contrast. As shown in Fig. 2D, DA/Ag_4_-H exhibited as a homogeneous sphere with no obvious AgNPs distributed in it, this may be due to the fact that the silver grains are not formed much and the grain size is too small to be clearly observed. While DA/Ag_7_-H and DA/Ag_10_-H were in obvious contrast with the inner high-density nuclear shadow and the outer low-density shell shadow, exhibited a typical core–shell (i.e. silver–polydopamine) structure [24, 42], which is consistent with the analysis results of the aforementioned XPS, WCA and ICP-MS. Interestingly, no obvious silver particles were detected for DA/Ag_4_-H, we speculate that the silver was homo-dispersed in the polydopamine matrix with the monovalent state or reduced small silver cluster. This important feature endowed the DA/Ag_4_-H surface with better performance in the following biological experiment. Besides, the size of NPs gradually decreased with the increase of reacting pH, but the agglomeration gradually increased, which is consistent with SEM results.

**Figure 3.**
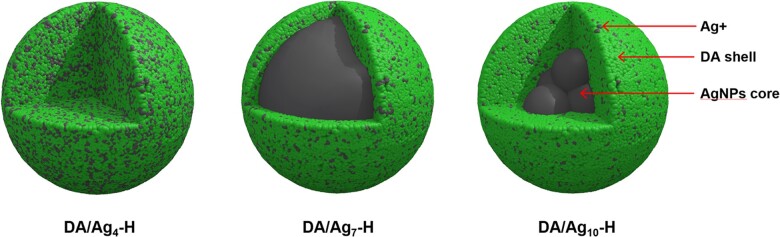
The proposed structures of DA/Ag nanocomposites that prepared under acidic (pH = 4), neutral (pH = 7) and alkaline (pH = 10) conditions. The DA/Ag4-H is structurally homogenous with most Ag^+^ distributed in it, whereas both DA/Ag7-H and DA/Ag10-H have typical core (AgNPs) and shell (polydopamine) structures, and the shell of DA/Ag10-H is thicker and its core is smaller.

The change of size distribution with reaction time was investigated by dynamic light scattering (DLS). As shown in Fig. 2E, at the first 1 h of reaction, the average sizes of particles that were prepared under different pH were all < 500 nm with little differences. At the second hour, the particles at pH 7 and pH 10 had slightly smaller diameter than 500 nm, while particles at pH 4 kept the diameter around 200 nm. From the third hour, the sizes ranked in ascending order as follows: pH 4 < pH 7 < pH 10. After 24 h of reaction, the particle sizes were 492.5 **±** 64.6 nm for DA/Ag_4_-H, 1121.7 **±** 297.8 nm for DA/Ag_7_-H and 2614.2 **±** 305.9 nm for DA/Ag_10_-H, respectively. Compared with TEM measurements, the particle size measured by DLS is larger, which may be caused by aggregation between particles, so these data are more suitable for measuring the particle formation rate. The particles size in pH 10 solution showed the most rapid growth, while that of pH 7 moderately grew and that of pH 4 remained basically stable. The zeta potential of particles is a key indicator of the stability of a particle dispersion. Figure 2F exhibited the zeta potential at different time points. The zeta potential of particles at pH4 remained positive values within 24 h, which was the reason for its good dispersibility. The potential at pH 7 and pH 10 were negative values till 6 h and 12 h, respectively, when the potential became positive values. At 12 h and 24 h, the particles in all solutions were positively charged and the potential value at pH 4 was higher than that at pH 7 and pH 10. The driving forces for the growth of AgNPs include the gradual growth of a single nucleus and the agglomeration of multiple primary nuclei (silver atom clusters) into large particles [29]. During the growth of AgNPs, there is always dynamic encapsulation by solute molecules (DA or oligomers). The surface potential of AgNPs is crucial to its dispersibility. Under acidic conditions, the polymerization of DA was slow, and it did not have enough driving force to reduce Ag^+^ into large AgNPs, so the silver particles were fixed in the particles as invisible silver nanoclusters or silver ions. On the contrary, higher reaction pH would lead to the formation of a large number of silver nuclei in the initial stage, then underwent an Ostwald ripening in the following stage, eventually produced the larger AgNPs tending to achieve a thermodynamically stable state [43]. Based on this, we propose the structures of DA/Ag nanocomposites prepared at different pH as shown in Fig. 3.

### *In vitro* antibacterial assessment

During implantation, bacteria in the mouth could latch onto the implant surface or invade the implant site through the crevice between the implant and surrounding tissues, leading to infection or inflammation (peri-implant inflammation and peri-implant mucositis). Therefore, antibacterial implants should inhibit bacterial adhesion and biofilm formation on the implant surface and meantime inhibit the bacteria in surrounding tissues to avoid infection-associated inflammation. *Staphylococcus aureus*, one of the most common cause of implant infection [44], could colonize onto the implant surface within 30 min after implant placement [6], and initial *S. aureus* colonization may contribute to the adhesion and colonization of other bacteria, which make for the development of peri-implant lesions [45].

**Figure 4.**
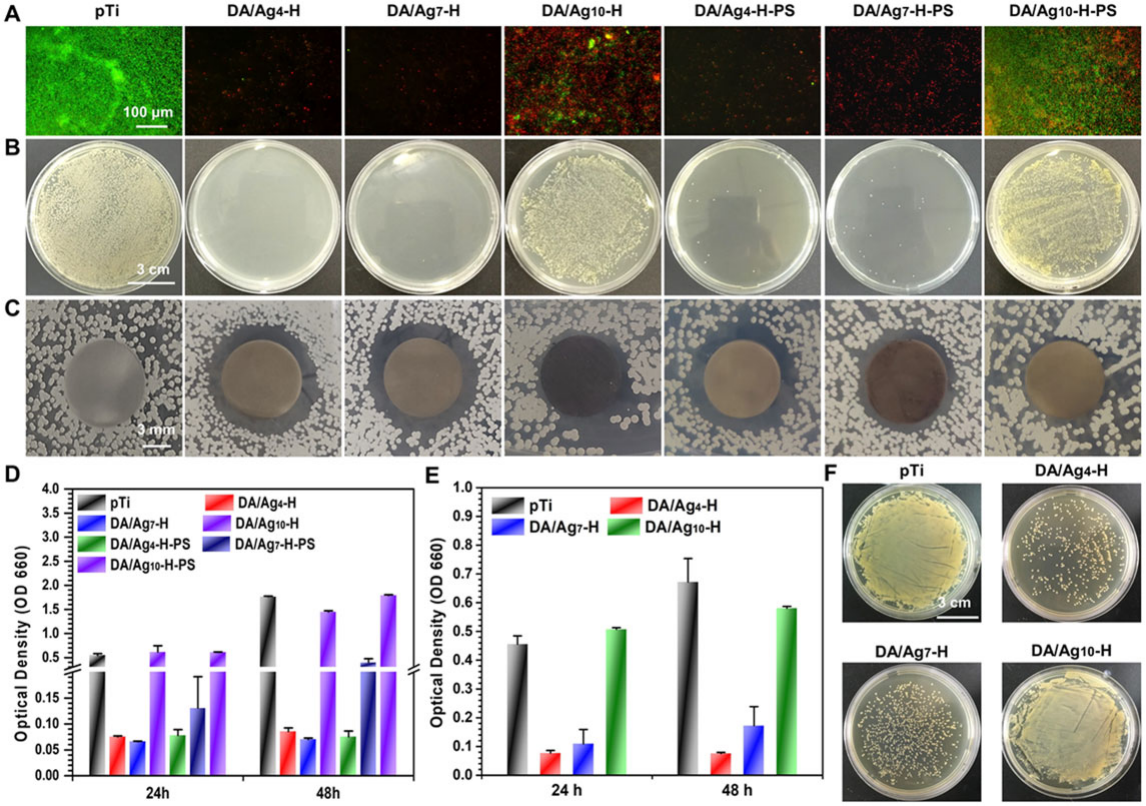
(**A**) Live/dead staining of *S. aureus* on different sample surfaces, and (**B**) the photographs of colonies cultured on agar plates, and the (**C**) zone of inhibition (ZOI) test of different samples. (**D**) Antibacterial efficiency of different samples tested by bacterial concentration (positive correlation with OD value) in LB medium. Antibacterial efficiency of the used samples was tested again by (**E**) bacterial concentration in LB medium and (**F**) colony density on agar plate.

Live/dead bacteria staining and the spread plate method were used to evaluate the ability of the prepared surfaces to inhibit bacterial adhesion or kill adhesion bacteria. Here, *in situ* evaluation of the adhered *S. aureus* on the surfaces was performed by fluorescently staining live/dead bacteria. As shown in Fig. 4A, bacteria adhered rapidly and extensively to the surface of pTi, while most of the adhered bacterial cells were alive (stained green). Among the DA/Ag-modified surfaces, the surfaces of DA/Ag_4_-H and DA/Ag_7_-H almost completely inhibited bacterial adhesion and killed adhesion bacteria. The spread plate method was used to observe the number of live *S. aureus* adhered to the surface. As shown in Fig. 4B, there were also no live bacteria on the surfaces of DA/Ag_4_-H and DA/Ag_7_-H, and a large number of live bacteria adhered onto the surfaces of pTi and DA/Ag_10_-H.

Here, the inhibition ring method (Fig. 4C) and the turbidimetric method (Fig. 4D) were used to evaluate the abilities of the samples to inhibit the surrounding bacteria. Clear transparent inhibition zone was observed around DA/Ag_4_-H and DA/Ag_7_-H, showing the release antibacterial property of these samples. The samples were incubated with bacterial suspensions of *S. aureus*, and then the bacterial quantity measured by the turbidimetric method with optical density at 660 nm (OD 660) showed that the samples of DA/Ag_4_-H and DA/Ag_7_-H also exhibited excellent antibacterial activity.

Before implant bone integration is accomplished, bacteria may invade the implant site through the crevice. Studies have shown that 1 week is necessary for soft tissue initial sealing to prevent bacteria from invading crevice [46], so the DA/Ag-modified samples immersed in PS for 7 days were used to evaluate the stability of the surface antibacterial ability by the above methods. The DA/Ag_10_-H-PS still displayed no antibacterial ability. The samples of DA/Ag_4_-H-PS and DA/Ag_7_-H-PS also showed strong antibacterial activity, but the antibacterial activity of DA/Ag_7_-H-PS was slightly weakened. While compared with the samples without immersion in PS, the incubated DA/Ag_4_-H still show strong antibacterial property. In addition, in order to evaluate the effectiveness of the antibacterial properties of the samples after repeated use, the tested samples were washed and dried, and the antibacterial evaluation was performed again. As shown in Fig. 4E and F, the used samples of DA/Ag_4_-H and DA/Ag_7_-H also demonstrated good antibacterial activity, and the antibacterial activity of DA/Ag_4_-H was superior to that of DA/Ag_7_-H, which was consistent with the antibacterial results of the samples immersed in PS for 7 days. There were sporadic live bacteria on the surface of DA/Ag_4_-H, and there was a large number of live bacteria adhered to other surfaces. These results also showed that the surfaces of DA/Ag_4_-H had a good ability to inhibit bacterial adhesion or kill bacteria, and the stronger antibacterial activity may closely relate to the large amount of Ag^+^ exposed on the outermost surface of DA/Ag_4_-H. This experiment also verified that the DA shell made it difficult for the silver core to be effectively released into the surrounding environment, thereby greatly weakening its antibacterial properties.

### *In vivo* antibacterial assessment

**Figure 5.**
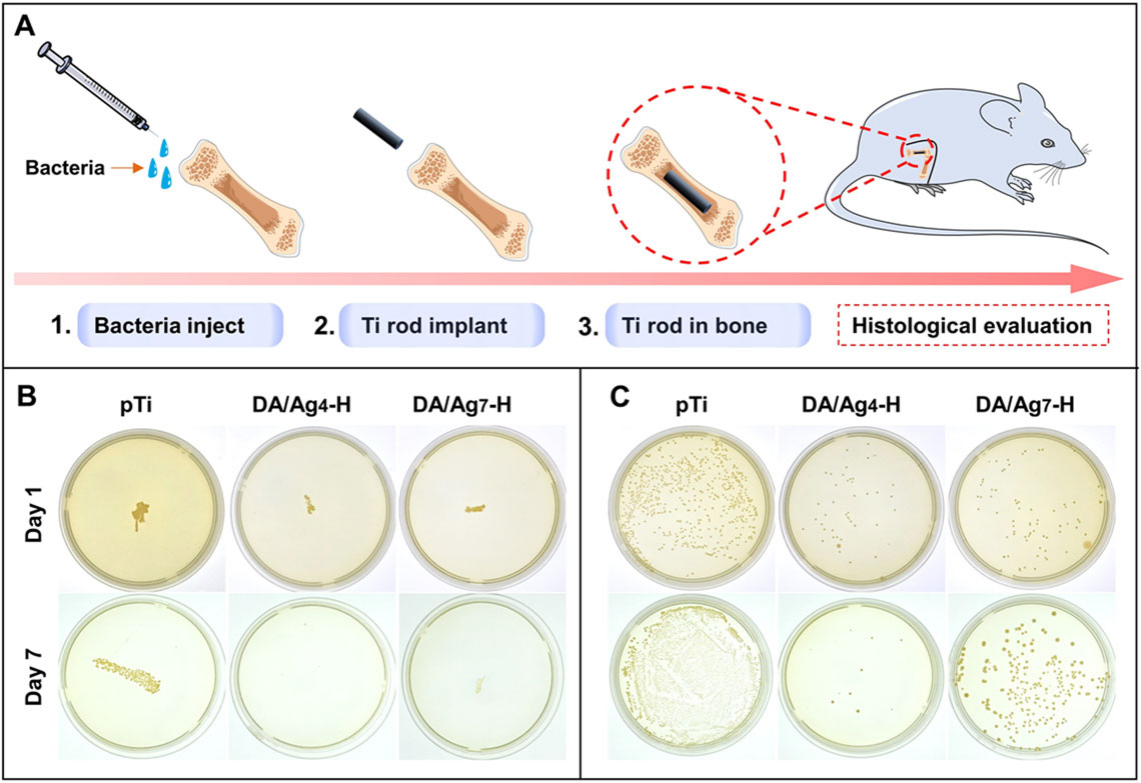
(**A**) Schematic diagram of the *in vivo* evaluation of the antibacterial properties of DA/Ag nanocomposites coated titanium rods. Antibacterial assay of different samples against *S. aureus* after 1 day and 7 days implantation of the rods in the femoral medullary cavity by (**B**) roll-over and (C) spread plate culture of the colonies.

There are more than 700 kinds of bacteria in the human oral cavity, and bacterial infection will seriously affect the success rate of implant surgery. The titanium rod with pre-soaked bacterial solution was implanted into the femoral pulp cavity of the rat model to simulate the antibacterial ability of the sample in the bacteria-containing alveolar bone species, thus to evaluate the real antibacterial performance of the modified material in the living environment (Fig. 5A). Because the *in vitro* antibacterial results showed that DA/Ag_4_-H and DA/Ag_7_-H had good antibacterial ability, these samples were further evaluated for *in vivo* antibacterial effects. After 1 and 7 days of implantation, the samples were removed and rolled on agar to be cultured for another 24 h. As shown in Fig. 5B, after 1 day of implantation, a large number of bacteria adhered to the titanium rod surface, and the number of bacteria that were adhered to the surfaces of DA/Ag_4_-H and DA/Ag_7_-H was significantly less than that of the pTi surface. In addition, the bacteria adhered onto the samples were detached by ultrasound for the spread plate test. The results showed that the number of bacterial colonies of pTi, DA/Ag_7_-H and DA/Ag_4_-H decreased successively (Fig. 5C), which was consistent with that of the rolling culture. After 7 days of implantation, the results of rolling culture and spread plate method showed that the number of adherent bacteria on the surface of pTi and DA/Ag_7_-H increased significantly compared with that of 1 day, but the number of bacteria on DA/Ag_7_-H surface was still much less than that on the pTi surface. In addition, bacteria were almost completely inhibited or killed on DA/Ag_4_-H surface, illustrating that the antibacterial effect of this surface *in vivo* could be more obvious with the extension of implantation time.

### Cytocompatibility

**Figure 6.**
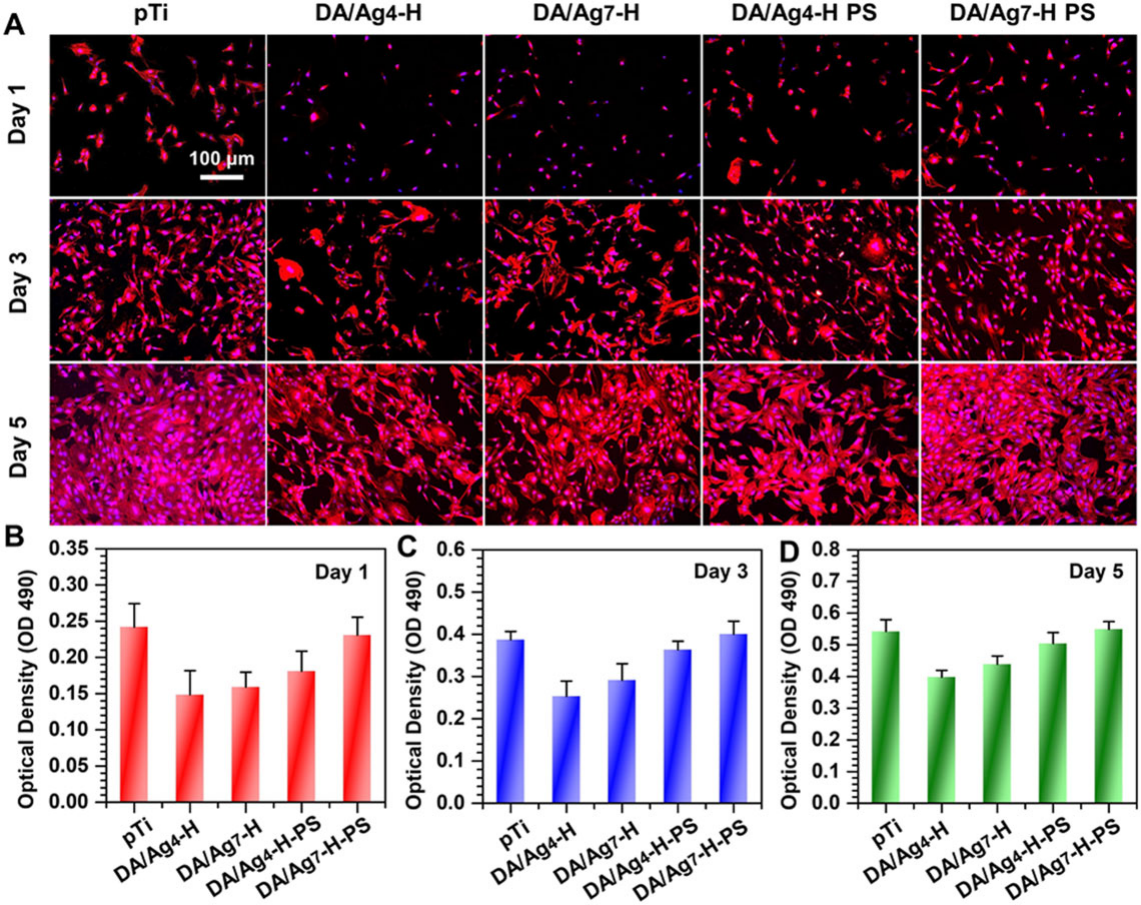
(**A**) Fluorescence images (YF555-phalloidin for F-actin and DAPI for nuclei) and MTT assays of MC3T3-E1 cells cultured for (**B**) 1, (**C**) 3 and (**D**) 5 days on different samples.

The antibacterial surface is considered to be a short-term method to inhibit bacteria-related complications because the antibacterial substance on the surface is gradually embedded or released, leading to a decline in antibacterial ability. To ensure a long-term therapeutic effect, it is very important to achieve rapid osseointegration with alveolar bone at the bone–implant interface and soft tissue integration at the transmucosal region to seal the crevice between the implant and surrounding tissues to prevent bacterial invasion [46]. Therefore, cell fluorescence staining and MTT assays were used to evaluate the biocompatibility of the samples with the mouse osteoblastic cell line (MC3T3-E1). As shown in Fig. 6A, the results of fluorescence staining showed that there was no significant difference in the number of adherent cells on the different surfaces after 1 day of culture, but the difference in cell spreading was very obvious, and cell spreading on the pTi surface was the best, and that on the surfaces of DA/Ag_4_-H and DA/Ag_7_-H was the worst. After 3 days of culture, all samples showed significant cell proliferation, almost all the cells on the surfaces of pTi and DA/Ag_7_-H-PS showed good spread and healthy morphology, meanwhile, some cells on the other three surfaces did not spread or showed abnormally large sizes, especially DA/Ag_4_-H and DA/Ag_7_-H. After 5 days of culture, the surfaces of pTi and DA/Ag_7_-H-PS were almost completely covered by spindle-shaped cells. Most of the surface area of the other three samples was also covered by cells, while only the cells on the DA/Ag_7_-H-PS surface showed a healthy spinning shape, demonstrated that PS treatment could effectively reduce the cytotoxicity of DA/Ag_4_-H. We speculated that this may be due to the large amount of Ag^+^ removed from the surface by PS. The result of MTT assay showed that cell activity on all surfaces increased with the prolongation of culture time. But the values of cell viability of DA/Ag_4_-H and DA/Ag_7_-H was significantly lower than that of pTi at 1, 3 and 5 days (Fig. 6B–D, respectively), indicating that these surfaces have certain cytotoxicity. However, there were no significant difference of the cell viability among DA/Ag_4_-H-PS,DA/Ag_7_-H-PS and pTi after 5 days of culture, implied that the cytotoxicity of DA/Ag_4_-H and DA/Ag_7_-H could decrease significantly with the increase of implantation time.

The surface of DA/Ag_4_-H has the best antibacterial activity in all prepared surfaces, but it also showed a strong cytotoxicity at the first day of culture, and however, the cytotoxicity was attenuated with prolonged incubation, especially in PS-treated samples. Compared to that of pTi, the relative growth rate (RGR) of DA/Ag_4_-H was 61.4, 65.4 and 73.4% after 1, 3 and 5 days culture, respectively. While in a previous study in which AgNPs were directly exposed to polydopamine coatings, osteoblasts on this surface had an RGR of only 36.7% after 5 days [15]. These results indicate that DA/Ag_4_-H with a homogeneous structure endows the coating with good antibacterial properties and improved cytocompatibility by reducing the direct exposure of silver on the surface. DA/Ag_4_-H-PS cultured for 5 days. The RGR was even increased to 93%, with almost no cytotoxicity. These results implying that the PS-treated DA/Ag_4_-H can greatly reduce the cytotoxicity or even be non-toxic by eluting the silver on the surface of the nanocomposite.

### Bacteria–cell co-culture test

**Figure 7.**
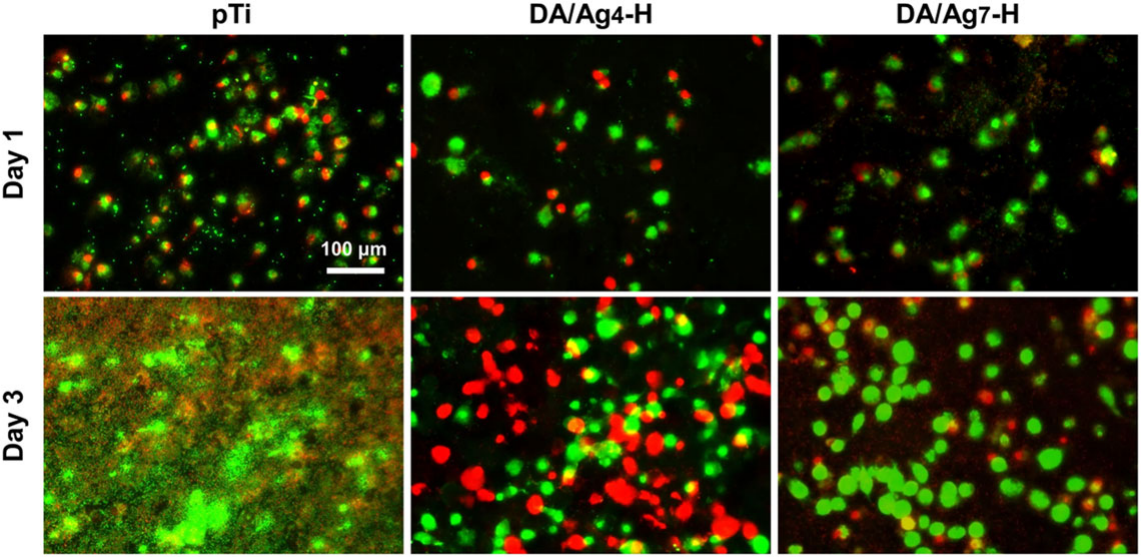
Live/dead staining of *S. aurues* and MC3T3-E1 that co-cultured for 1 and 3 days.

As the oral cavity is rich in bacteria, host cells and bacteria need to simultaneously compete for implant surface colonization after implantation [47]. When host cell integration to an implant surface could reduce bacterial contamination, bacteria preferentially colonize the material surface, resulting in an inability of host tissue–implant integration to out-compete bacterial adhesion and growth, which further results in bacterial-related complications. Therefore, researchers have proposed that better progress can be gained by developing infection-resistant surfaces to both inhibit bacterial adhesion and promote tissue integration [48]. Here, considering that the rapid adherence of *S. aureus* to implant surface may happen within 30 min after dental implant surgical operation [6], the samples inoculated with *S. aureus* were co-cultured with osteoblasts. As shown in Fig. 7, after 1 day of culture, the fluorescence images showed a large number of osteoblasts and *S. aureus* (small dots) on pTi, and some cells and sporadic bacteria adhered on DA/Ag-modified surfaces. After 3 days of culture, adherent bacteria covered the pTi surface completely and no normal morphological cells were observed, whereas there were a large number of osteoblasts on the DA/Ag-modified surfaces with little observable bacteria. The number of dead cell (in red) on DA/Ag_4_-H is slightly higher than that of DA/Ag_7_-H. These results demonstrated that in the cell–bacteria competition environment around implants, bacteria adhesion and growth could be effectively suppressed by DA/Ag_4_-H and DA/Ag_7_-H, and the adhesion and growth of osteoblast were not influenced too much by the silver in both the two nanocomposites. Compared with silver core/DA shell in DA/Ag_7_-H, the structurally homogeneous DA/Ag_4_-H could expose more silver ions well on the outermost surface, resulting in stronger antibacterial ability and cytotoxicity. Although the proportion of viable cells on the surface of DA/Ag7-H was higher, its antibacterial performance was poor and probably led to the difficulty in effectively inhibiting the inflammatory response of bacteria. As a matter of fact, when designing an implant surface, an appropriate balance between antibacterial activity and osseointegration is of great difficulty and necessity [49, 50]. The surfaces of DA/Ag_4_-H and DA/Ag_7_-H attained the preliminary balance to some extent, especially the DA/Ag_4_-H.

### Subcutaneous implantation and femoral intramedullary implantation *in vivo*

**Figure 8.**
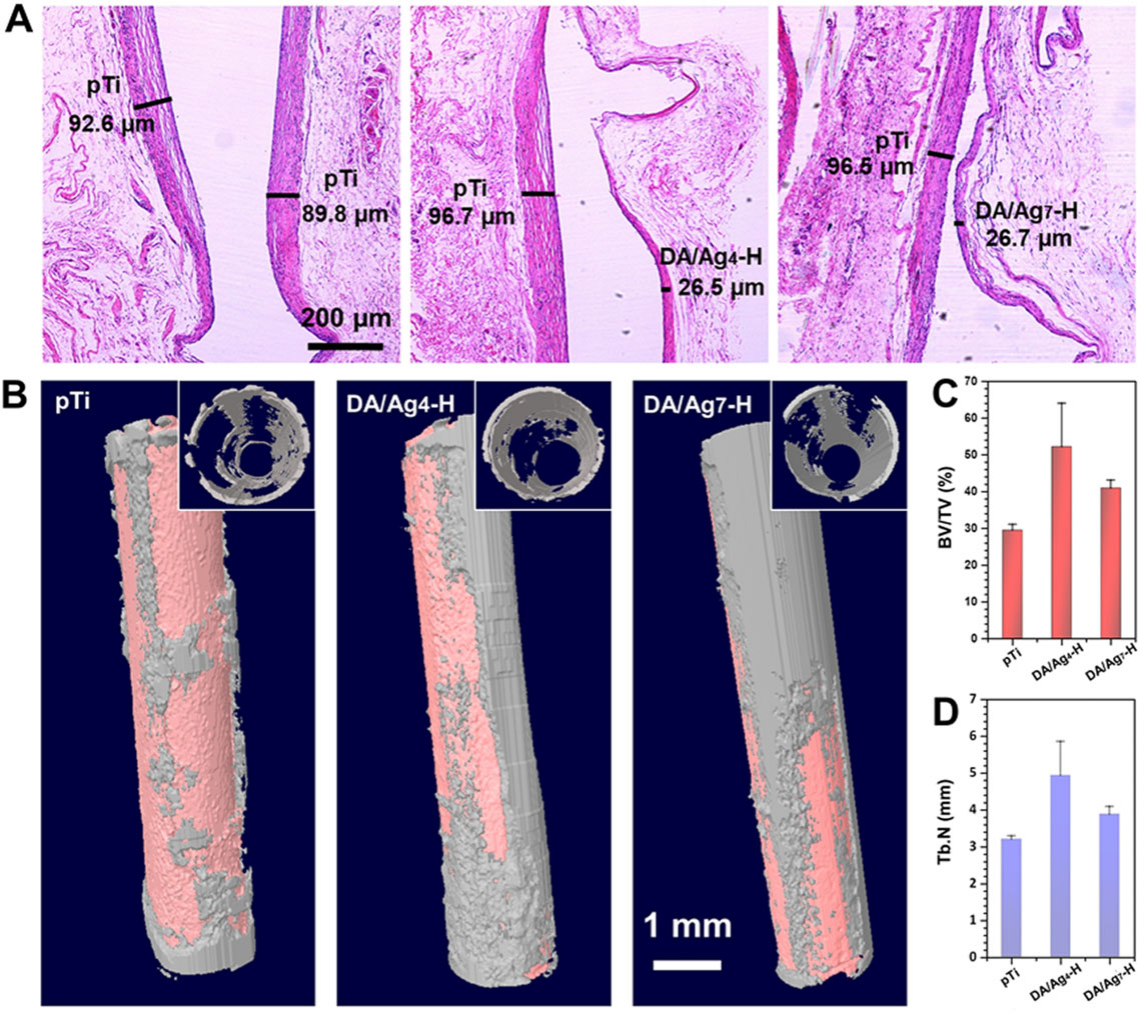
(**A**) Histological sectioning (HE staining) of subcutaneous tissues around the bare and coated titanium implants after 1 month implantation. The thickness of fibrous cyst wall at the back (pTi) and the modified side (pTi, DA/Ag4-H and DA/Ag7-H) of each sample were measured. (**B**) 3D micro-CT reconstructed images of the new bone formation around the implanted sample rods (the inset in each group is a view along the Central axis of the rod). Quantitatively assessing of (**C**) the bone volume to tissue volume (BV/TV) and (**D**) trabecular number (Tb.N) values.

The soft tissue at the transmucosal region of the implant is easily invaded by bacteria and causes peri-implant mucositis, which forms deep periodontal pockets and impedes bone integration. Therefore, materials were implanted into the subcutaneous tissue of rats with bacteria to assess tissue responses in this region and evaluate the antibacterial surface to inhibit bacteria-induced inflammation. After 1 month of implantation, the tissue around the materials was harvested, and then HE staining for histological examination was carried out, which is shown in Fig. 8A. As a marker of soft tissue inflammation, the fibrous capsules thickness around pTi, DA/Ag_4_-H and DA/Ag_7_-H were 88.2 ± 20.7 μm, 23.6 ± 10.6 μm and 27 ± 8.4 μm, respectively, indicating that the DA/Ag-modified surfaces had a stronger inhibitory effect on the initial inflammation caused by microorganisms after implantation, preventing capsule formation. Some studies have reported that lipopolysaccharides secreted by bacteria can stimulate macrophages and lead to inflammatory responses; thus, antibacterial surfaces can alleviate inflammation and promote soft tissue healing by inhibiting bacteria in the oral microenvironment [46]. The process of wound healing renders the environment advantageous to bacterial colonization and growth [6], and the accumulation of plaque will induce the inflammation of peri-implant soft tissues [51]. In previous *in vitro* antibacterial assessment, we simulate the eluting of nanocomposite coating in physiological environment by immersing the samples in PS, demonstrated that the coating maintained good antimicrobial properties and improved cytocompatibility over time. As expected, the *in vivo* test showed that the inflammatory response was effectively reduced by both DA/Ag_4_-H and DA/Ag_7_-H.

Integration with alveolar bone is essential for long-term stability of dental implants. To investigate the *in vivo* osteointegration ability of DA/Ag-modified surfaces under infected conditions, micro-CT was performed to evaluate the peri-implant of new bone formation in the femoral medullary cavity after 1 month of implantation. As shown in Fig. 8B, new bone was observed around all the samples at 4 weeks. The statistics of (Fig. 8C) bone volume to tissue volume and (Fig. 8D) trabecular number values revealed that the new bone mass was ordered as follows: DA/Ag4-H > DA/Ag7-H > pTi. These results demonstrated that DA/Ag-modified samples (especially the DA/Ag4-H) significantly enhanced new bone formation at the early stage of osteointegration in the infected animal model by likely avoiding the over-activation of the immune system [52]. Osteogenesis experiments further demonstrated that DA/Ag nanocomposite with a homogeneous structure could achieve optimal osteogenic properties by regulating the balance of antibacterial efficiency and cytocompatibility.

## Conclusion

In this study, a simple method for preparing structurally diverse DA/Ag nanocomposites was developed to modify the surface of titanium implants. By simply regulating the pH of the mixed solution of DA and silver nitrate and subsequent heat treatment, the DA/Ag nanocomposites were firmly bonded to the titanium surface. DA/Ag nanocomposites that were prepared under acidic (pH = 4) condition are structurally homogenous with highest concentration of Ag^+^, whereas the nanocomposites prepared under neutral (pH = 7) and alkaline (pH = 10) conditions presented the typical core (AgNPs) and shell (polydopamine) structures.

###  

Besides, the insufficient polymerization of the DA component in the homogeneous DA/Ag nanocomposite (DA/Ag4-H) made the DA/Ag4-H degraded faster, leading to the efficient release of Ag^+^, thus showing stronger and longer-lasting antibacterial properties. Although the optimized DA/Ag4-H still had a slight cytotoxicity at the initial stage of application, it would significantly attenuate the degradation of nanocomposites; thus it had a little adverse effect on the extended cytocompatibility test. Cell–bacterial co-culture *in vitro* and long-term subcutaneous implantation and femoral medullary cavity implantation test in the bacterial environment revealed that the structurally homogenous DA/Ag_4_-H surface has the best comprehensive outcomes with better antibacterial efficiency and less cytotoxicity. This study provides a new idea to overcome the challenges of simultaneously realizing the good antibacterial performance and favorable cytocompatibility by fabricating a homogeneous DA/Ag nanocomposite coating on titanium dental implants.

## Supplementary data

Supplementary data are available at *Regenerative Biomaterials* online.

## Supplementary Material

rbac082_Supplementary_Data
