# Supplementary material for: A homogeneous dopamine–silver nanocomposite coating: striking a balance between the antibacterial ability and cytocompatibility of dental implants
Source: Regen Biomater. 2022 Oct 20;10:rbac082. doi: 10.1093/rb/rbac082 (PMC9847628; doi:10.1093/rb/rbac082)
Supplement: rbac082_Supplementary_Data [file rbac082_supplementary_data.zip › rbac082_Supplementary_Data/Supporting infomation.docx]

*Supporting information*

A homogeneous dopamine-silver nanocomposite coating: Striking a balance between the antibacterial ability and cytocompatibility of titanium implants

Shuang Wang^a,^**^†^**, Zichen Wu^a,^**^†^**, Yankai Wang^a,^**^†^**, Huilei Hong^a^, Lijie Zhang^a^, Zhaoyang Chen^a^, Pengkang Zhang^a^,Weibo Zhang^a^, Shunli Zheng ^a^, Quanli Li^a^, Wei Li^a,*^, Xiangyang Li^a,*^, Hua Qiu^a,*^, Jialong Chen^a,*^.

^a^ Stomatologic Hospital and College, Anhui Medical University, Key Laboratory of Oral Diseases Research of Anhui Province, Hefei, Anhui 230032, China

**^†^** These authors contributed equally to this work

* Corresponding authors. E-mail addresses: 453951372@qq.com (W. Li), qiuhua1990@foxmail.com (H. Qiu), hlxiangyang@163.com (X. Li), jialong_dt@126.com (J. Chen).


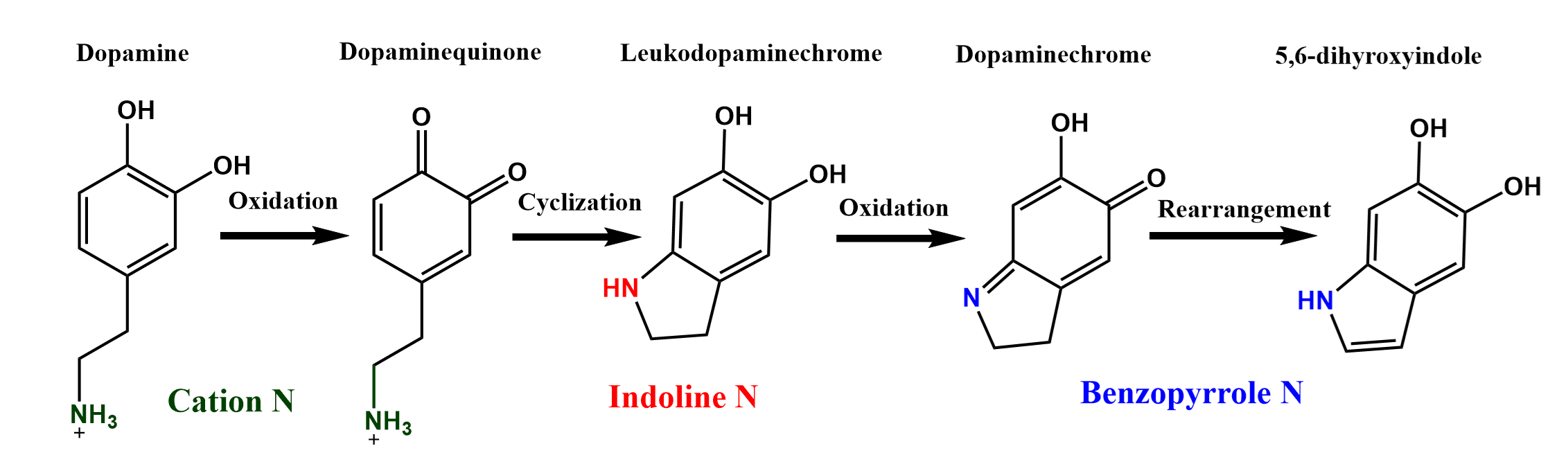


**Figure S1**. The products of dopamine in different stages of oxidation reaction and the chemical state of nitrogen in it. Note that protonation of dopamine amino groups occurs under acidic conditions.

**Table S1.** The area proportion of assigned components

| **Samples** | **Peak assignment** | | |
| --- | --- | --- | --- |
|  | **Indoline N (%)** | **Benzpyrole N (%)** | **Cation N (%)** |
| **DA/Ag_4_** | 36.23 | 5.05 | 58.72 |
| **DA/Ag_7_** | 48.95 | 26.02 | 25.03 |
| **DA/Ag_10_** | 49.27 | 45.28 | 5.45 |
| **DA/Ag_4_-H** | 54.55 | 8.18 | 37.27 |
| **DA/Ag_7_-H** | 67.6 | 23.28 | 9.12 |
| **DA/Ag_10_-H** | 70.21 | 26.44 | 3.35 |


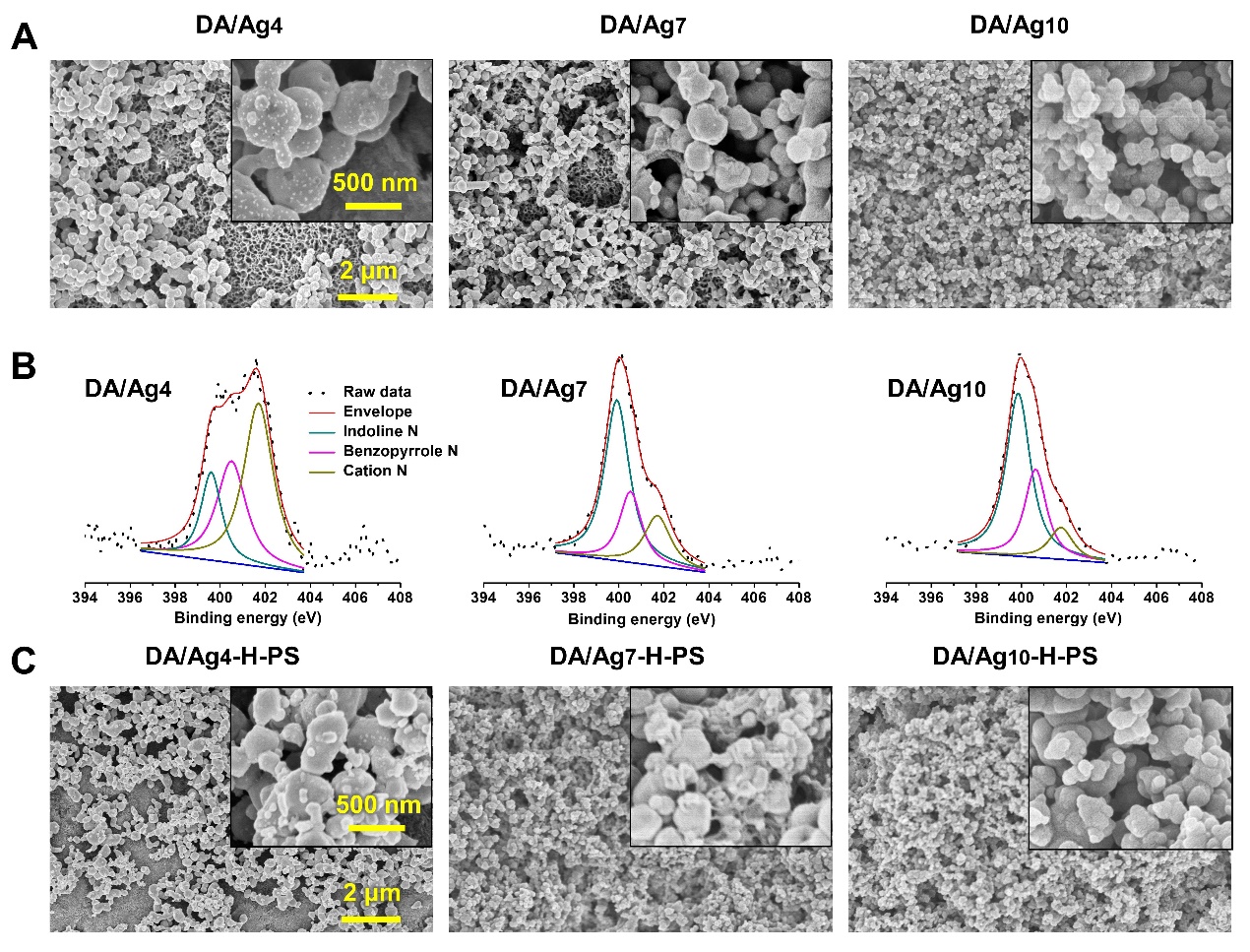


**Figure S2.** (A) Surface morphology of DA/Ag4, DA/Ag7 and DA/Ag10 nanocomposite coatings by SEM. (B) The high-resolution spectra of N 1s and the fitted peaks of Indoline N, Benzopyrrole N and Cation N for each sample. (C) Surface morphology of DA/Ag4-H-PS, DA/Ag7-H-PS and DA/Ag10-H-PS nanocomposite coatings after immersed in physiological saline for 7 days.
