# Supplementary material for: A homogeneous dopamine–silver nanocomposite coating: striking a balance between the antibacterial ability and cytocompatibility of dental implants
Source: Regen Biomater. 2022 Oct 20;10:rbac082. doi: 10.1093/rb/rbac082 (PMC9847628; doi:10.1093/rb/rbac082)
Supplement: rbac082_Supplementary_Data [file rbac082_supplementary_data.zip › rbac082_Supplementary_Data/Response to reviewer.docx]

**Reviewer: 1**

Comments to the Author

The manuscript entitled “A homogeneous dopamine-silver nanocomposite coating: Striking a balance between the antibacterial ability and cytocompatibility of dental implants” provided a view on the relationship between material structure and biological function. It has certain value in overcoming the conflict between cytocompatibility and antibacterial properties that exists widely in silver-containing antibacterial materials. I think only some minor problems should be addressed:

1.The format of the full text should be standardized. In line 7, page 8, “1 mL” is highlighted in red and should be changed to black font. In line 47, page 8, and in line 6, page 10, the citation symbols are inconsistent with the rest of the text.

2.The histogram data of the full text have bars with statistical variance except for Figure 2C, . Are the statistics of silver volume reliable enough?

3.Some grammar mistakes need to be checked and the author should better to ask a native English speaker to polish the manuscript.

Response to reviewer 1:

1) The format of the full text has been checked again and standardized.
2) Thanks for pointing out our mistakes. The silver content was detected on parallel samples. However, we ignored the error bar when plotting. We have made corrections to Figure 2C.
3) We have corrected some grammatical errors in the text, and the changes are highlighted in yellow in the revised manuscript.

**Reviewer: 2**

Comments to the Author

1. It is recommended that more statement of the instructive meaning to the implant dentistry could be added to the introduction or discussion part, in order to emphasize more about the clinical significance of this research. And it is also recommended to introduce more relevant studies for comparison and enrich the discussion in the “results and discussion” section. This could enhance the reference value of the study.

2. Page13，Line22-27：In this paragraph the results in FigS2C on the immersion test were described, but from line 22 to 27 the correlation between antibacterial results and the particle structure was suddenly discussed. I consider the discussion here is not appropriate, given that the antibacterial results are firstly presented in the part 3.2, and the evidence is still not enough to support the “homogeneous” and “dopamine shell” structure before the following other results are shown. I would suggest discussing this correlation after the concept of “homogeneous” and “core-shell” structure are put forward and antibacterial results are presented.

3. There are various formatting problems or spelling errors in the text, such as unusually red words of "1 ml" in line 49 on page 7 and in line 8 on page 8. Besides, there are irregular citation formats in the text. Some citation numbers are superscripts, while some are square brackets. This needs to be unified as required.

4. In Figure 7, the picture of bacterial cell co-culture is not clear, please adjust the resolution of the picture.

5. A couple of grammar mistakes should be corrected, such as: “with” after “attenuate” should be deleted in line 4 on Page 26; “that” after “Ag+” should be deleted in the second line from the bottom on Page17; “however” should be modified to “and however” to connect the two sentences in line 2 on Page21; “may lead to the difficult to ...” in line 19-20 on Page 22 should be modified to something like “probably led to the difficulty in doing sth” or “probably rendered/made it difficult to do sth”.

Response to reviewer 2:

1) The reviewer indicated that more statement of the instructive meaning to the implant dentistry could be added to the introduction or discussion part. Our opinion is that the relationship between implant surface infection and failure is succinctly and comprehensively explained by citing adequate literatures in the first paragraph of the introduction, and then the necessity of surface antibacterial is pointed out. We don't think it's necessary to exhaustively describe implant failures in multiple paragraphs, which would make the article seem lengthy. However, the recommendation of introducing more relevant studies for comparison and enrich the discussion in the “results and discussion” section is quiet a good suggestion. Unfortunately, although there are many studies on antibacterial dopamine-silver coatings, there are few studies directly applied the DA/Ag to dental implants, and we have cited and discussed these papers in the text.
2) Thank the reviewer’s valuable suggestion. The antibacterial results appearing here are a bit abrupt. In view of the similar results and discussions we had presented in section 3.2, we just delete the antibacterial discussion here.
3) Thank the reviewer for carefully reviewing this article. Formatting problems and grammar mistakes have been corrected in the revised manuscript. The changes are highlighted in yellow.
4) We have checked the original images in figure 7. It looks a bit blurry due to exposure control and intentionally high contrast, but we don't think it affects the analysis of the results.

**Reviewer 3**

Comments to the Author

1.Please check the spelling and grammar, for example, the authors wrote “was determined through S. aureus inhibition test osteoblast-like MC3T3-E1 cells test, respectively. ” There should be an “and” between “inhibition test” and “osteoblast-like MC3T3-E1”. All “S. aureus” should be Italic.

2.Materials Part: “in male SD rats weighing about 200 mg”. How can a rat be 200 mg?

3.The authors mentioned “However, applying PDA-AgNPs to device surfaces still faces two major

challenges. One is how to make PDA-AgNPs firmly bonded to the surface, which is important to

resist frictional shedding during implantation”. Please provide evidence that the novel methods for preparing silver coating showed greater adhesion force on implant surface.

4.The authors indicated that “(even promoting) the osseointegration.”, please provide the possible reason for this.

5.How did the authors determine the heating temperature.

6.The authors should also provide the bone formation result in bone marrow without infection, as not all implant insertion is accompanied by bacterial infection. Thus, the titanium shouldn’t impact bone formation on titanium surface under normal conditions.

7. The authors evaluated S.aureus as model bacteria, which is not a regular habitant in oral cavity.

Response to reviewer 3:

1) Thank the reviewer for carefully reviewing this article. We have checked the spelling and grammar in the revised manuscript, the changes are highlighted in yellow. In the materials part,“in male SD rats weighing about 200 mg” has been corrected to “in male SD rats weighing about 200 g”.
2) Thank the reviewer for valuable suggestion. It is necessary to firmly attach nanoparticles to implants for clinical application. In the pre-experimental stage, we have tried to verify the mechanical stability of the nanoparticle coating. We simulated the clinical application by pressing the coating on the pork bone piece and rubbing it repeatedly, and then evaluated the antibacterial properties of the rubbed coating by bacterial inhibition ring test (**Fig. 1**). For the DA/Ag4-H and DA/Ag7-H groups, the results showed that their antibacterial effect was enhanced after the rubbing test. We speculate that the rubbing caused more silver to be exposed on the surface. While the antibacterial effect of the DA/Ag10-H group was similar before and after rubbing, which may be due to the thick dopamine shell preventing core silver from exposure. The above results **were not** as expected (i.e. rubbing caused the coating to peel off and the antibacterial property was weakened, by which we could indirectly evaluate the stability of the coating). Therefore, this method may not be suitable for evaluating the coating stability, so these results are not presented in the manuscript. Instead, we investigated the coating stability through other methods, such as determination of the resistance to ultrasonic cleaning of the coating, repeated antibacterial testing of the used coating to verify its durability of antibacterial performance, and borrowing from proven methods [1][2] to improve the stability of dopamine particles, etc. We believe these methods could prove the stability of the coating to some extent, please understand.

**Figure 1**. Schematic illustration of rubbing test and the antibacterial ability of the rubbed coatings.

3) The reviewer asked us to provide the possible reason for “(even promoting) the osseointegration”, that’s really a good idea. Whether the “promotion” is real or not depends on the experimental reference. In fact, we do not think that implants with antimicrobial coatings have an advantage in promoting osteogenesis compared to uncoated titanium in uninfected models. However, this study is aimed at an infection-prone situation after implantation, so we used an infectious animal model by injecting bacterial fluid around the implant, and verified that the coated implants enhanced bone formation in this case. In the Results and Discussion section, we also gave the possible reasons and cited the related reference: *"These results demonstrated that DA/Ag modified samples (especially the DA/Ag4-H) significantly enhanced new bone formation at the early stage of osseointegration in the infected animal model by likely avoiding the over-activation of the immune system [3]"*. However, for the rigor of the conclusions and to avoid possible confusion for the reader, we have decided to delete the word "promoting" in the revised manuscript.
4) Heating temperature is determined based on our experience and previous research [1][2]. The researchers studied the thermal stability of pDA by TGA (**Fig. 2a**) and TGA-MS (**Fig. 2b**), and accordingly they selected the temperature of 130 °C for the heat treatment of pDA. We increased the processing temperature to 150°C within a reasonable temperature window. Under this temperature, the pDA coating was dehydrated and undergoes further cyclization of oligomers and more ordered and tight rearrangement of the sub-nanoscale structure, ultimately resulting in higher mechanical strength and swelling resistance.

**Figure 2**. The stability of pDA. (a) TGA curve of the pDA showing more than 90% mass retention up to the annealing temperature of 150 ˚C. (b) TGA-MS spectrum of the pDA indicating that most of the mass loss at lower temperatures (below 150℃) can be attributed to the dehydration [2].

5) As for the reviewer's suggestion to perform a bone formation assay in an uninfected animal model, this question is partially answered in the third answer. Some patients who need dental implants have periodontal diseases, and bacterial colonies may exist locally. In addition, there are a large number of bacterial flora in the oral cavity, this increased the risk of implant related infections. However, the healthy rats we used in this study do not have the above problems, and the implant-associated infections are rare under normal surgical conditions. The operated rats can usually achieve good osseointegration due to their excellent self-healing capacity. Therefore, the osteogenesis effect of our antibacterial coating can only be tested by deliberately injecting bacterial fluid to simulate the infected microenvironment around the implants. Some previous studies have also performed similar experiments in this way [4][5].
6) The oral flora is complex, with typical representatives such as streptococcus mutans，porphyromonas gingivalis，staphylococcus，lactobacillus and anaerobes. Some flora in peri-implantitis, such as *S. aureus*, are able to adhere to titanium surfaces tightly and play an important role in early bacterial adhesion and mediating bacterial biofilm maturation [6][7], some high-level studies exploring the influence of bacteria on the osseointegration of titanium surfaces also used *S. aureus* [8].

**Reference:**

[1] Proks, V., Brus, J., Pop-Georgievski, O., Večerníková, E., Wisniewski, W., Kotek, J., Urbanová, M. Rypáček. Thermal-Induced Transformation of Polydopamine Structures: An Efficient Route for the Stabilization of the Polydopamine Surfaces. *Macromolecular Chemistry and Physics* 2013; 214: 499-507.

[2] Malollari KG, Delparastan P, Sobek C, Vachhani SJ, Fink TD, Zha RH, Messersmith PB. Mechanical Enhancement of Bioinspired Polydopamine Nanocoatings. *ACS Appl Mater Interfaces* 2019; 11: 43599-607.

[3] Silvia Spriano, Seiji Yamaguchi, Francesco Baino, Sara Ferraris. A critical review of multifunctional titanium surfaces: New frontiers for improving osseointegration and host response, avoiding bacteria contamination. *Acta Biomaterialia* 2018; 79:1-22

[4] M Lucke, G Schmidmaier, S Sadoni, B Wildemann, R Schiller, N.P Haas, M Raschke. Gentamicin coating of metallic implants reduces implant-related osteomyelitis in rats. *Bone* 2003; 32:521-531.

[5] Liping Ouyang, Yaochao Zhao, Guodong Jin, Tao Lu, Jinhua Li, Yuqin Qiao, Congqin Ning, Xianlong Zhang, Paul K. Chu, Xuanyong Liu. Influence of sulfur content on bone formation and antibacterial ability of sulfonated PEEK. *Biomaterials* 2016; 83:115-126,

[6] Aguayo S, Donos N, Spratt D, Bozec L. Nanoadhesion of staphylococcus aureus onto titanium implant surfaces. *J Dent Res* 2015; 94:1078-1084.

[7] Mirjam M. Fürst, Giovanni E. Salvi, Niklaus P. Lang, G. Rutger Persson, G. Rutger Persson. Bacterial colonization immediately after installation on oral titanium implants. *Clin Oral Implants Res* 2007; 8:501-508

[8] Guodong Jin, Hui Qin, Huiliang Cao, Yuqin Qiao, Yaochao Zhao, Xiaochun Peng, Xianlong Zhang, Xuanyong Liu, Paul K. Chu. Zn/Ag micro-galvanic couples formed on titanium and osseointegration effects in the presence of S. aureus. *Biomaterials* 2015; 65:22-31.

**Review Editor**

Comments to the Author:

Preferred font type of the texts in the GA is Arial 8-16 points (smaller will not be legible).

Response to review editor:

We have changed the fonts in GA to be larger, but if it is larger than 8 points, the fonts in the figure will appear crowded or even overlapped, so some fonts are smaller than 8 points.
